# Descriptions of three new *carbonaria*-group species of *Fannia* Robineau-Desvoidy from China, with a key to the *carbonaria*-group species (Diptera, Fanniidae)

**DOI:** 10.3897/zookeys.657.9153

**Published:** 2017-02-17

**Authors:** Ming-fu Wang, Wei Li, Yu-wan Zhao, Jie Wu, Dong Zhang

**Affiliations:** 1 Institute of Entomology, Shenyang Normal University, Shenyang, 110034, P.R. China; 2 College of Biological Sciences and Biotechnology, Beijing Forestry University, Beijing, 100083, P.R. China; 3 Shanghai Entomological Museum, Chinese Academy of Sciences, Shanghai, 200032, P.R. China; 4 School of Nature Conservation, Beijing Forestry University, Beijing, 100083, P.R. China; 5 Key Laboratory in Healthy Science and Technology, Division of Life Science, Graduate School at Shenzhen, Tsinghua University, Shenzhen, 518055, P.R. China

**Keywords:** *Fannia carbonaria*-group, Fanniidae, identification key, new Chinese species

## Abstract

A historical review of the *Fannia carbonaria*-group is provided and three new species are described from China: *Fannia fani* Wang & Wu, **sp. n.**, *Fannia nitidiventris* Wang & Zhang, **sp. n.** and *Fannia submaculata* Wang & Zhao, **sp. n.**. One species, *Fannia norvegica* Ringdahl, 1934, is recorded for the first time from China. Illustrations of male terminalia of these four species and a taxonomic key to the males of known species in the group are given. The *Fannia carbonaria*-group now includes 30 species distributed in the Holarctic Region and northern part of the Oriental Region.

## Introduction

The *Fannia carbonaria*-group is one of the species-groups of genus *Fannia*. It was established by Chillcott (1961) for 15 species arranged in two subgroups, namely the *Fannia carbonaria*-subgroup and the *Fannia minutipalpis*-subgroup. Species of this group are characterized by the following character states: mid tibia usually with two anterodorsal setae and two posterodorsal setae; hind femur without a posteroventral seta at distal part; and thorax usually with one stout prealar seta.

Since the 19^th^ century, a number of investigators have turned their attention to what is now termed the *Fannia carbonaria*-group, including Meigen (1826), Verrall (1892), Stein (1895, 1920), Ringdahl (1934), D’Assis-Fonseca (1966), Nishida (1974a, 1974b, 1975a, 1975b) and Gregor and Rozkošný (1993). Studies at the end of 1900s added eight European species to the group (consisting of five species in the *Fannia carbonaria*-subgroup and three in the *Fannia minutipalpis*-subgroup; Rozkošný, Gregor & Pont, 1997), as well as five Chinese species (Xue and Wang 1998).

At the beginning of 21^st^ century, Nishida (2002, 2003) studied the Japanese species of the *Fannia carbonaria*-group. Wang, Xue and Su (2004) revised eight species in the group from China. Wang et al. (2009) reviewed the cosmopolitan fauna of the *Fannia carbonaria*-subgroup by updating a key to males, presenting a diagnosis to each species, and describing two new species from China. Recently, study of Barták et al. (2016) found out that *Fannia lucida* Chillcott, 1961 is a junior synonym of *Fannia norvegica* Ringdahl, 1934. This brought the total number of known species of the *Fannia carbonaria*-group to 27, containing 17 species in the *Fannia carbonaria*-subgroup and ten in the *Fannia minutipalpis*-subgroup.

During a study of the Fanniidae fauna, three new species belonging to the *Fannia carbonaria*-subgroup were discovered in China and are described herein. The total number of species in the *Fannia carbonaria*-group is now 30. Illustrations of male terminalia are given for the species and an updated key to the identification of males is provided.

## Materials and methods

Terminology follows McAlpine (1981) and Stuckenberg (1999). Absolute measurements in millimeters (mm) are given for body length. All type specimens of the new species are deposited in the Institute of Entomology, Shenyang Normal University, Shenyang, China (**IESNU**) except for those of *Fannia fani* sp. n., which are deposited in the Shanghai Entomological Museum, Chinese Academy of Science, Shanghai, China (SHEM). Methods for the preparation of terminalia and illustrations follow Zhang et al. (2013). Abbreviations used throughout the text are as follow:

acr
 acrostichal seta,

ad
 anterodorsal seta,

av
 anteroventral seta,

d
 dorsal seta,

dc
 dorsocentral seta,

ia
 intra-alar seta,

p
 posterior seta,

pd
 posterodorsal seta,

pra
 prealar seta,

pv
 posteroventral seta.

## Taxonomic accounts

### Key to the males of the *Fannia carbonaria*-group

**Table:** 

1	Hind coxa bare on posterior surface; palpus normal (*Fannia carbonaria*-subgroup)	**2**
–	Hind coxa with setulae on posterior surface; palpus short (*Fannia minutipalpis*-subgroup)	**21**
2	Hind femur swollen on posteroventral surface in distal half	**3**
–	Hind femur not swollen on posteroventral surface in distal half	**4**
3	Presutural *acr* triserial; katepisternal setae 0+1; mid coxa with 3–8 *ad*; hind femur with 6 or 7 stout *av* with tips of setae curved on swollen part; cercal plate with cluster of slender setae in upper part	***Fannia xiaoi* Fan** [China]
–	Presutural *acr* biserial; katepisternal setae 1+1; mid coxa without *ad*; hind femur with only 2 or 3 *av* with tips of setae not curved on swollen part; cercal plate without cluster of setae in upper part	***Fannia fani* Wang & Wu, sp. n.** [China]
4	Haltere dark brown or black	**5**
–	Haltere yellowish or brownish yellow	**7**
5	*Pra* 2; hind femur with 8 or 9 *pv* in basal half	***Fannia subfuscitibia* Wang** [China]
–	*Pra* 1; hind femur without distinct *pv*	**6**
6	Presutural triserial; calypters yellowish; *pra* 3/4 to 4/5 as long as posterior notopleural seta; cercal plate without median apical process	***Fannia corvina* Verrall** [China, Japan, North America, throughout Europe]
–	Presutural biserial; calypters brown; *pra* 1/2 as long as posterior notopleural seta; cercal plate with median apical process	***Fannia maculosa* Nishida** [Japan]
7	Hind femur without distinct *pv*	**8**
–	Hind femur with distinct *pv*	**16**
8	Wing hyaline	***Fannia borealis* Chillcott** [Canada]
–	Wing distinctly yellow or brown	**9**
9	Calypters white; haltere yellowish	***Fannia fuscitibia* Stein** [Czech Republic, Great Britain, Japan, throughout North America]
–	Calypters yellowish or yellow; haltere yellow or brownish yellow	**10**
10	Lower calypter not projecting beyond upper one and smaller	***Fannia melanura* Chillcott** [Throughout North America]
–	Lower calypter projecting beyond upper one and larger	**11**
11	Abdomen without pollinosity or stripe and therefore black and shining	***Fannia nitidiventris* Wang & Zhang, sp. n.** [China]
–	Abdomen with pollinosity and median linear stripe or triangular stripe	**12**
12	Syntergite 1+2 to tergite 4 each with a median dark linear stripe	**13**
–	Syntergite 1+2 to tergite 4 each with a triangular stripe	**15**
13	Presutural *acr* triserial	***Fannia dorsovittata* Wang** [China]
–	Presutural *acr* biserial	**14**
14	*Pra* stout, 3/4 length of posterior notopleural seta; hind tibia with 4 *ad*	***Fannia submaculata* Wang & Zhao, sp. n.** [China]
–	*Pra* short, 1/2 length of posterior notopleural seta; hind tibia with 3 or 4 *ad*	***Fannia urbana* Nishida** [Japan]
15	Mid tibia with 2 *ad*, 1 or 2 *pd*	***Fannia carbonaria* Meigen** [Canada, China, Japan, throughout Europe, United States]
–	Mid tibia with 3 *ad*, 3 or 4 *pd*	***Fannia pseudonorvegica* D’Assis-Fonseca** [Czech Republic, England, Hungary]
16	Parafacial with setae	***Fannia vernalis* Nishida** [Japan]
–	Parafacial bare	**17**
17	Hind femur with a complete *pv* row	**18**
–	Hind femur with *pv* only in basal 1/2 to 3/4	**20**
18	Hind femur with *pv* row becoming gradually weaker towards apex	***Fannia garretti* Chillcott** [Canada, United States]
–	Hind femur with *pv* row not becoming gradually weaker towards apex	**19**
19	Occipital setae present; hind tibia with 5–8 *ad*	***Fannia fulgida* Nishida** [Japan]
–	Occipital setae absent; hind tibia with 2 or 3 *ad*	***Fannia norvegica* Ringdahl** [China, Czech Republic, Denmark, Great Britain, Greek, Japan, North Africa, Norway, Spain, Switzerland, throughout North America]
20	*Pra* 1, stout, 2/3 to 3/4 length of posterior notopleural seta; mid tibia with 2 *ad*; hind tibia with 3 *ad* and 2 *av*	***Fannia imperatoria* Nishida** [China, Japan]
–	*Pra* 2, weak, the longest one 1/2 length of posterior notopleural seta; mid tibia with 1 *ad*; hind tibia with 1 *ad* and 1 *av*	***Fannia pallidibasis* Pont** [Morocco]
21	Syntergite 1+2 to tergite 4 each with a distinctly median dark triangular stripe	**22**
–	Syntergite 1+2 to tergite 4 each with a median linear stripe or an inverted T-shaped linear stripe	**26**
22	Mid tibia with 1 *ad*	***Fannia capricornis* Xue** [China]
–	Mid tibia with 2 or 3 *ad*	**23**
23	Occipital setae in complete row; frontal setae 7–9	***Fannia neopolychaeta* Chillcott** [North America]
–	Occipital setae with gap in row in posterior 1/3; frontal setae 9–13	**24**
24	In ventral view, surstylus not projecting at middle on posterior margin, bacilliform sclerite curved and hook-like in distal part	***Fannia trigonifera* Chillcott** [United States]
–	In ventral view, surstylus projecting and horn-like and becoming broader at middle on posterior margin, bacilliform sclerite straight in distal part	**25**
25	Mid femur with 6–8 long and sparse *av* in basal 2/3	***Fannia japonica japonica* Nishida** [Japan]
–	Mid femur with only short *av* in basal half	***Fannia japonica amamiensis* Nishida** [Japan]
26	Palpus at most 1/2 length of prementum	**27**
–	Palpus more than 1/2 length of prementum	**28**
27	Hind tibia with only 1 stout *ad*; palpus less than 1/2 length of prementum	***Fannia minutipalpis* Stein** [China, Czech Republic, Germen, North America, Slovak Republic]
–	Hind tibia with 2 stout *ad*; palpus 1/2 length of prementum	***Fannia brevipalpis* Chillcott** [United States]
28	Parafacial at middle 2/3 as wide as width of postpedicel	***Fannia pauli* Pont** [Europe, Russia]
–	Parafacial at middle 1/3–1/2 as wide as width of postpedicel	**29**
29	Hind femur with *pv* row in basal 2/3 to basal part; postpedicel three times longer than wide	***Fannia tauricornis* Wang, Xue & Su** [China]
–	Hind femur with *pv* row only in basal 1/2 to basal part; postpedicel 2.5 times longer than wide	**30**
30	Eye with sparse and short hairs; prementum with thin gray pollinosity; *pra* 3/4 length of posterior notopleural seta	***Fannia polychaeta* Stein** [Austria, Czech Republic, Germany, Russia, Sweden; Bermuda]
–	Eye bare; prementum black and shining, without pollinosity; *pra* 2/3 length of posterior notopleural seta	***Fannia antilocera* Wang, Xue and Su** [China]

### Descriptions of three new species from China

#### Fannia fani

Taxon classificationAnimaliaDipteraFanniidae

Wang & Wu sp. n.

http://zoobank.org/1A7E9708-AFB6-45ED-948E-EB35BAF6C084

Fig. 1

##### Diagnosis.

This species is characterized as follows: presutural *acr* biserial; katepisternal setae 1+1; calypters yellowish; haltere yellow; mid coxa without *ad*; mid femur without ventral spine; mid tibia with only one *d* and one *v* at apex; hind femur with only two or three stout *av* on swollen part in distal half, the longest one not longer than 1/2 of hind tibial length, all other short hair-like; cercal plate without long setae cluster in upper part.

**Figure 1.**
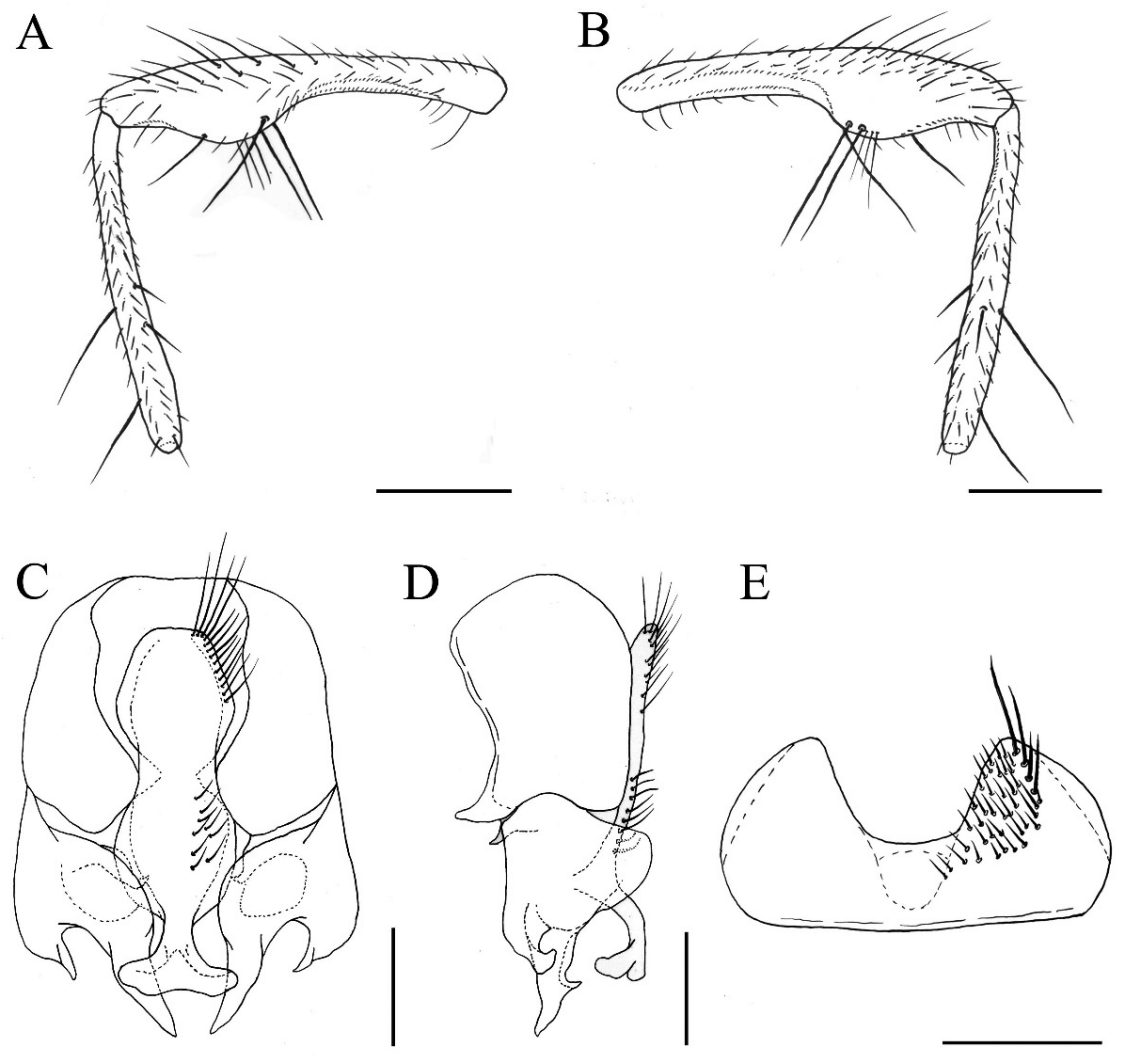
*Fannia fani* Wang & Wu, sp. n., male, holotype (specimen from Heilongjiang, deposited in SHEM). **A** Right hind leg, anterior view **B** Right hind leg, posterior view **C** Terminalia, ventral view **D** Terminalia, lateral view **E** Sternite 5, ventral view. Scale bars: **A–B** 0.50 mm; **C−E** 0.25 mm.

##### Description.

MALE. Body length 4.5−5.0 mm. Eye with sparse and short light brown hairs; upper inner facets larger than the rest; postocular setae in one row, long and fine, curved anteriorly in the upper part of head, occipital setae behind the postocular setae on vertex; fronto-orbital plate and parafacial with grayish silver pollinosity; at narrowest point frons slightly wider than the distance between outer margins of two posterior ocelli, as wide as the width of postpedicel; frontal stripe black, with thin gray pollinosity, at narrowest point slightly narrower than the width of fronto-orbital plate; frontal setae 12–15, stout, nearly reaching ocellar triangle; without orbital seta; parafacial bare, at middle as wide as the width of postpedicel; antenna black, postpedicel 1.6 times longer than wide, arista black; epistoma not projecting beyond vibrissal angle, vibrissal angle behind frontal angle in profile; subvibrissal setulae in one row, lateral of subvibrissal setulae with some fine setae; gena and genal dilation with black setulae, upper margin of gena with upcurved setae; prementum with thin grayish yellow pollinosity, 2.5 times longer than wide; palpus black, claviform, as long as prementum. Thorax black in ground color, notum with brownish gray pollinosity, and with four slightly wide but indistinct stripes; presutural *acr* biserial, only prescutellar pairs slightly stout, the distance between *acr* rows 1/2 of the distance between *acr* row and *dc* row, *dc* 2+3, *ia* 0+2, *pra* 1, 3/5 to 2/3 of the length of posterior notopleural seta, notopleuron without setula; proepisternal setae 2, proepimeral setae 2, around proepimeral setae with ten to 13 slender setulae; basisternum, proepisternum, anepimeron, meron and katepimeron bare; katepisternal setae 1+1, katepisternum without ventral spine; anterior spiracle yellowish, small, posterior one yellow; calypters yellowish, the lower one slightly projecting beyond the upper one. Wing brownish; veins brown; tegula dark brown; basicosta yellowish brown; costal spine inconspicuous; node of Rs bare on ventral and dorsal surfaces; vein R_4+5_ straight, vein M_1+2_ slightly close to vein R_4+5_ distally; crossveins without obvious cloud; halter yellow but brown in distal part. Legs entirely black; fore coxa without anterior spine on ventral surface, fore femur with complete *pv* row, fore tibia without *ad* and *p*; mid coxa without a hook-like spine or spine-like seta, mid femur with long and sparse *av* in basal part, becoming gradually denser and shorter towards apex, biserial and short spine-like in preapical part, then with a gap towards apex and with four or five comb-like setae in distal part, *ad* row complete but short, *pv* row complete and stout, biserial in preapical part, behind *pv* row with a complete and stout row of setae, mid tibia slightly slender in basal 2/5 and slightly swollen in distal 3/5, with one *ad* and one or two *pd* in distal half, with one *d* and one *v* at apex, and with numerous slender setulae on ventral surface, the longest one slightly shorter than mid tibial width in distal part, mid first tarsomere without basal tooth-like spine on ventral surface; hind coxa bare on posterior surface, hind femur slightly curved and swollen in distal half, with *av* only on swollen part, 2-3 of them stout, other trichia all short hair-like, *ad* row stout (Fig. 1A), posterior to posteroventral surface bare in basal 2/3, with five *pv* in distal 1/3, only two slightly stout, with three or four slender *pv* in distal part (Fig. 1B), hind tibia with one median *av*, without *ad*, with one stout median *d*, with one *d* in distal half (Fig. 1A, B). Abdomen long and flattened, black in ground color, with gray pollinosity; syntergite 1+2 to tergite 4 each with a median triangular stripe, stripe on tergite 2 slightly broader in basal half, 1/2 as wide as the width of tergite, tergite 5 with a dark median stripe; sternite 1 with setulae, sternites 2 and 3 long and narrow, sternite 4 broad, sternite 5 strongly concave on posterior margin and straight on anterior margin, with four strong setulae above (Fig. 1E); cercal plate longish, broad in ventral view, slender in lateral view (Fig. 1C, D); surstylus broad at basal part, separated into two branches at middle, in lateral view the anterior one short and curved hook-like while the posterior one long and straight (Fig. 1C, D).

FEMALE. Unknown.

##### Remarks.

This new species appears to be most similar to *Fannia xiaoi* Fan, 2000 but differs by having *acr* in two rows; katepisternal setae 1+1; calypters yellowish; haltere yellow; mid femur without ventral spine; mid tibia with only one *d* and one *v* at apex; mid first tarsomere without any special structure; hind femur with only two or three stout *av* on swollen part in distal half, the longest one not longer than 1/2 of hind tibial length, all other short hair-like (Fig. 1A); cercal plate without long setae cluster in upper part (Fig. 1C, D).

##### Etymology.

The new species is named after Prof. Zi-de Fan in honor of his outstanding work on Calyptratae.

##### Type series.

Holotype male: China, Heilongjiang, Wuying, 12.V.1979, Coll. J. Shen (SHEM). Paratype: 1 male, the same data as holotype.

##### Distribution.

Known only from the type locality in Heilongjiang, China.

#### Fannia nitidiventris

Taxon classificationAnimaliaDipteraFanniidae

Wang & Zhang sp. n.

http://zoobank.org/B1D49BEA-B845-4047-BF0E-396CB099CD09

Fig. 2

##### Diagnosis.

This species is characterized as follows: frontal setae five or six; posterior *acr* biserial; calypters yellowish; haltere yellow; fore tibia all black; hind femur with three or four *av*; abdomen black and shining, without pollinosity or stripe; sternite 5 slightly concave on posterior margin; from ventral view, cercal plate slightly rounded, the hook-like projection on its lower margin curved outward; surstylus slightly broad.

**Figure 2.**
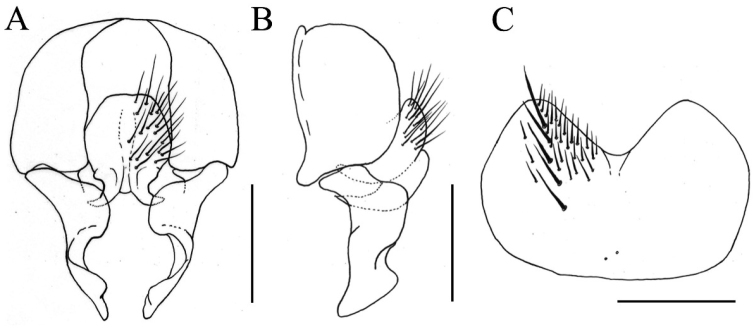
*Fannia nitidiventris* Wang & Zhang, sp. n., male, holotype (specimen from Ningxia, deposited in IESNU). **A** Terminalia, ventral view **B** Terminalia, lateral view **C** Sternite 5, ventral view. Scale bars 0.25 mm.

##### Description.

MALE. Body length 4.8 mm. The whole body shining black. Eye bare and large; postocular setae in one row, short and neatly arranged, without occipital seta; fronto-orbital plate and parafacial with gray pollinosity; at narrowest point frons 2/3 of the distance between outer margins of two posterior ocelli, 2/3 of the width of postpedicel; frontal stripe linear at narrowest point; frontal setae five or six, nearly reaching 2/3 of frons, the gaps filled with numerous fine setulae, orbital setae absent; parafacial bare and narrow, at middle as wide as 2/5 of the width of postpedicel; antenna black, postpedicel 2.0 times longer than wide, arista yellow in basal half, haired, the longest hair equal to basal aristal width; epistoma not projecting beyond vibrissal angle, vibrissal angle behind frontal angle in profile; subvibrissal setulae in one row, lateral of subvibrissal setulae row with a row of short setae; gena and genal dilation with black setulae, upper margin of gena without upcurved seta; proboscis stout, labella large; prementum shining, with thin grayish yellow pollinosity, 1.2 times longer than wide; palpus black, claviform, as long as prementum. Thorax black in ground color, shining, notum with thin grayish yellow pollinosity; *acr* biserial, hair-like, only prescutellar pairs stout, the distance between *acr* rows narrower than the distance between *acr* row and *dc* row, *dc* 2+3, *ia* 0+2, *pra* 1, 2/3 of the length of posterior notopleural seta, notopleuron without setula; proepisternal setae 2, proepimeral seta 1, lower part with one slender setula; basisternum, proepisternum, anepimeron, meron and katepimeron bare; katepisternal setae 1+1, katepisternum without ventral spine, with only some fine hair; anterior spiracle yellowish and small, posterior one brownish yellow; calypters yellowish, the lower one slightly projecting beyond the upper one. Wing brownish; veins brown; wing base being similar color as rest of wing; tegula black; basicosta brownish; costal spine inconspicuous; node of Rs bare on ventral and dorsal surfaces; vein R_4+5_ straight, veins R_4+5_ and M_1+2_ parallel to each other distally; crossveins without obvious cloud; haltere yellow. Legs entirely black; fore coxa without anterior spine on ventral surface, fore femur with complete *pv* row, fore tibia without *ad* and median *p*, with only one *d* in preapical part, fore first tarsomere with several longish basal setae on ventral surface; mid coxa without a hook-like spine or spine-like seta, mid femur with complete *av* row, short and strong, at middle the longest seta shorter than the width of mid femur, without gap, comb-like in distal 1/3, with complete *pv* row, biserial in median part, the longest one is shorter than the width of mid femur, with one fine *p* row, the longest one is equal to the width of mid femur, mid tibia slightly swollen towards apex, with three *ad*, two *pd*, and with numerous slender setulae on ventral surface, the longest one 2/3 of mid tibial width in distal half, mid first tarsomere without basal tooth-like spine on ventral surface; hind coxa bare on posterior surface, hind femur not curved, with three or four stout *av* only in distal 2/5, without *pv*, hind tibia with two *av*, two *ad* and one median *d.* Abdomen oval and flattened, black in ground color, shining, without stripe and pollinosity; sternite one bare, sternite 5 broad, concave on posterior margin (Fig. 2C); from ventral view, cercal plate slightly rounded, the hook-like projection on its lower margin curved outward (Fig. 2A); surstylus slightly broad, not separated into two branches (Fig. 2A, B).

FEMALE. Unknown.

##### Remarks.

The new species is distinguished from its likely closest relative *Fannia fuscitibia* Stein, 1920 by having five or six frontal setae; posterior *acr* biserial; calypters yellowish; haltere yellow; fore tibia all black; hind femur with three or four *av*; abdomen black and shining, without stripe; sternite 5 slightly concave on posterior margin (Fig. 2C); from ventral view, cercal plate slightly rounded, the hook-like projection on its lower margin curved outward (Fig. 2A); surstylus slightly broad (Fig. 2B).

##### Etymology.

This specific name refers to a characteristic of the species that the abdomen is black and shiny.

##### Holotype.

Male: China, Ningxia, Jingyuan, Lvyuan, 1700 m, 29.V.2008, Coll. M.F. Wang (IESNU).

##### Distribution.

Known only from the type locality in Ningxia, China.

#### Fannia submaculata

Taxon classificationAnimaliaDipteraFanniidae

Wang & Zhao sp. n.

http://zoobank.org/F9EB81F9-7C6A-4FD3-90FC-411FDA09C65F

Fig. 3

##### Diagnosis.

This species is characterized as follows: postsutural *acr* in two rows; *pra* stout, 3/4 of the length of posterior notopleural seta; calypters and haltere in lighter color; knees and base of fore tibia yellow; hind tibia with four *ad*; tergite 5 with a distinct dark median stripe; cercal plate slender in ventral view; surstylus with a distinct notch on inner margin in median part; bacilliform sclerite becoming gradually narrower towards apex.

**Figure 3.**
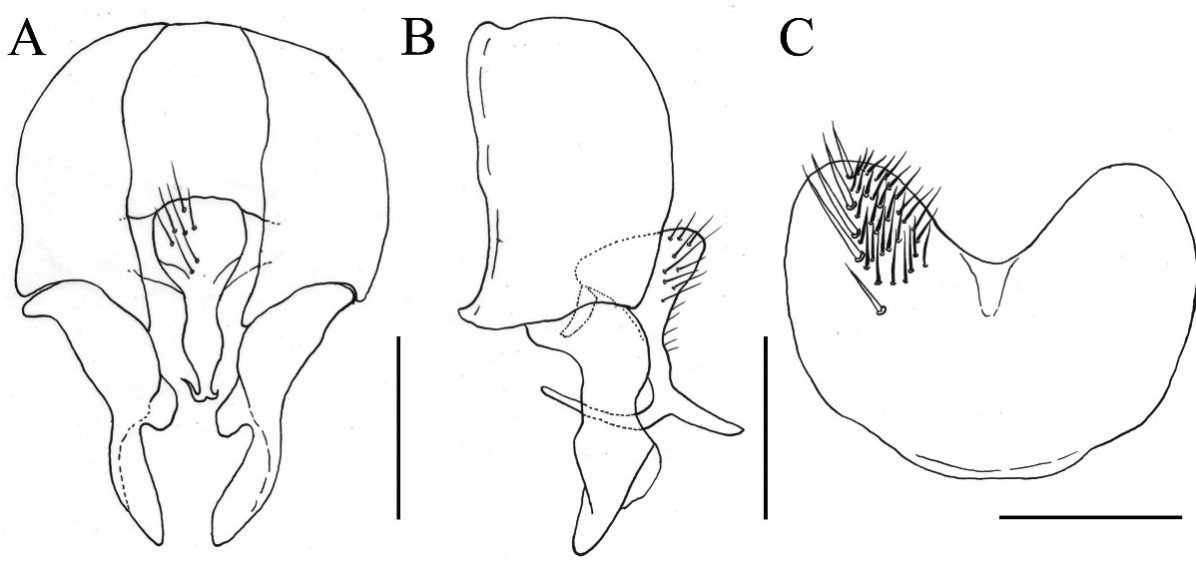
*Fannia submaculata* Wang & Zhao, sp. n., male, holotype (specimen from Ningxia, deposited in IESNU). **A** Terminalia, ventral view **B** Terminalia, lateral view **C** Sternite 5, ventral view. Scale bars 0.25 mm.

##### Description.

MALE. Body length 5.0 mm. Eye bare; upper inner facets larger than the rest; postocular setae in one row, irregular in length, curved anteriorly, with some occipital setae behind the postocular setae on vertex; fronto-orbital plate and parafacial with grayish white pollinosity; at narrowest point frons equal to the distance between outer margins of two posterior ocelli, equal to the width of postpedicel; frontal stripe black and narrow; frontal setae nine or ten all stout, situated on the lower 4/5 of fronto-orbital plate, nearly reaching ocellar triangle; parafacial bare, at middle 1/2 as wide as the width of postpedicel; antenna black, postpedicel 2.0 times longer than wide, arista haired and black, the longest hair equal to basal aristal width; epistoma not projecting beyond vibrissal angle, vibrissal angle behind frontal angle in profile; subvibrissal setulae in one row, gena and genal dilation with black setulae, upper margin of gena without upcurved seta; proboscis stout, labella large; prementum with grayish yellow pollinosity, 2.0 times longer than wide; palpus black, claviform, as long as prementum. Thorax black in ground color, shining, notum with thin brown pollinosity; *acr* biserial, slightly stout, prescutellar pairs stout; the distance between *acr* rows narrower than the distance between *acr* row and *dc* row, *dc* 2+3, *ia* 0+2, *pra* 1, 3/4 of the length of posterior notopleural seta, notopleuron without setula; proepisternal setae 2, proepimeral seta 1; basisternum, proepisternum, anepimeron, meron and katepimeron bare; katepisternal setae 1+1, katepisternum without ventral spine; anterior spiracle small and dark brown, posterior one dark brown; calypters yellowish, the lower one distinctly projecting beyond the upper one. Wing brownish; veins dark brown; wing base being similar color as rest of wing; tegula black; basicosta brownish; costal spine inconspicuous; node of Rs bare on ventral and dorsal surfaces; vein R_4+5_ straight, veins R_4+5_ and M_1+2_ parallel to each other distally; crossveins without obvious cloud; haltere brown. Legs entirely black, except knees and base of fore tibia yellow; fore coxa without anterior spine on ventral surface, fore femur with complete *pv* row, fore tibia without *ad* and median *p*, fore first tarsomere with several short basal setae on ventral surface; mid coxa without any hook-like spine or spine-like seta, mid femur with complete *av* row, stout in basal half, becoming gradually shorter and denser towards apex, without gap, *pv* row complete, in one row, with one slender *p* row, mid tibia slightly swollen in distal half, with two *ad*, two *pd*, and with numerous slender setulae on ventral surface, the longest one 3/4 as long as mid tibial width in distal part, mid first tarsomere without basal tooth-like spine on ventral surface; hind coxa bare on posterior surface, hind femur with *av* row, setula-like in basal half, with four long *av* in distal 2/5, without *pv*, hind tibia with two *av*, four *ad* and one median *d.* Abdomen oval and flattened, black in ground color, shining, with thin gray pollinosity; syntergite 1+2 to tergite 5 each with a dark median stripe; sternite one with slender setulae, sternite 5 broad, concave on posterior margin (Fig. 3C); cercal plate slender in ventral view, separated into two branches at apex, pointed anteriorly and posteriorly, respectively, in lateral view (Fig. 3A, B); surstylus with a distinct notch on inner margin at middle in ventral view (Fig. 3A); bacilliform sclerite short, becoming gradually narrower towards apex (Fig. 3B)

FEMALE. Unknown.

##### Remarks.

This new species is similar to *Fannia maculosa* Nishida, 2003 but can be characterised by its postsutural *acr* in two rows; *pra* long, 3/4 of the length of posterior notopleural seta; wing base being similar color as rest of wing; calypters and all haltere in lighter color; knees and base of fore tibia yellow; hind tibia with four *ad*; tergite 5 with a distinct dark median stripe; sternite 5 broad (Fig. 3C); cercal plate slender in ventral view (Fig. 3A); surstylus with a distinct notch on inner margin in median part (Fig. 3A); bacilliform sclerite becoming gradually narrower towards apex (Fig. 3B).

##### Etymology.

This specific name refers to the similarity between the new species and *Fannia maculosa* Nishida.

##### Holotype.

Male: China, Ningxia, Jingyuan, Dongshanpo, 2200 m, 27.VI.2008, Coll. M.F. Wang (IESNU).

##### Distribution.

Known only from the type locality in Ningxia, China.

### Redescription of one newly recorded species from China

#### Fannia norvegica

Taxon classificationAnimaliaDipteraFanniidae

Ringdahl, 1934

Fig. 4

##### Diagnosis.

This species is characterized as follows: parafacial bare; occipital setae absent; lower calypter distinctly projecting beyond upper one; haltere yellowish or brownish yellow; mid tibia with 2 *ad*; hind femur with a complete *pv* row, not becoming gradually weaker towards apex; hind tibia with 2 or 3 *ad*; syntergite 1+2 to tergite 4 each with a dark median triangular stripe, tergite 5 with a broad median stripe; sternite 5 broad and with four strong setulae; bacilliform sclerite curved.

**Figure 4.**
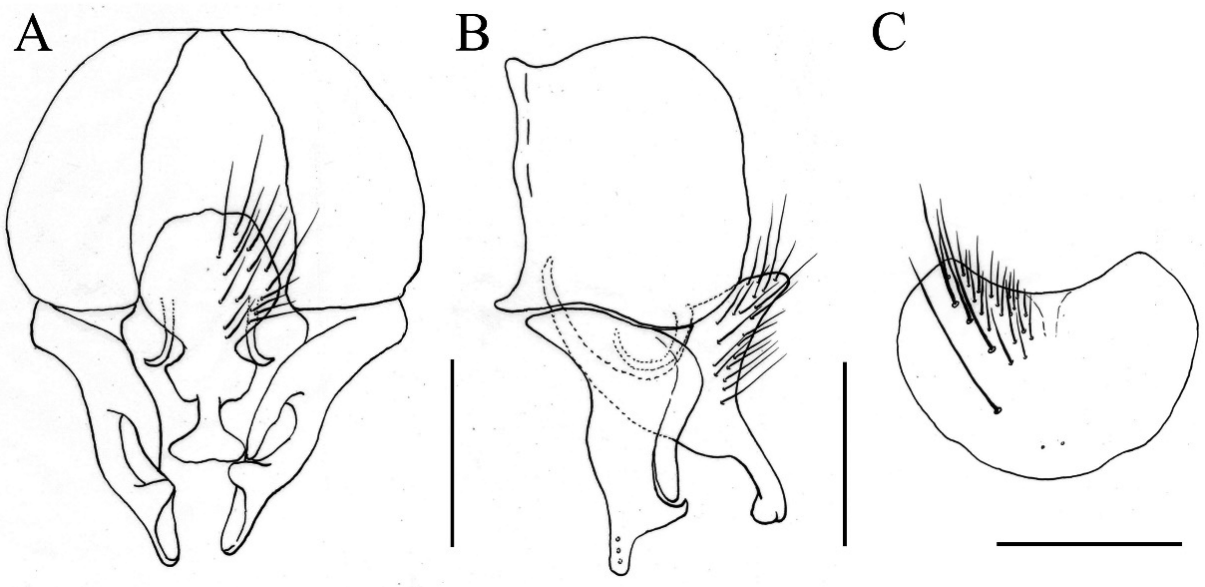
*Fannia norvegica* Ringdahl, 1934, male (specimen from Ningxia, deposited in IESNU). **A** Terminalia, ventral view **B** Terminalia, lateral view **C** Sternite 5, ventral view. Scale bars 0.25 mm.

##### Description.

MALE. Body length 4.8−5.5 mm. Eye bare; postocular setae in one row, as long as the width of frons, neatly arranged, without occipital seta; fronto-orbital plate and parafacial with grayish silvery pollinosity; at narrowest point frons as wide as the distance between outer margins of two posterior ocelli, equal to the width of postpedicel; frontal stripe black, at narrowest point as wide as fronto-orbital plate; frontal setae ten or eleven, stout, nearly reaching ocellar triangle, orbital setae absent; parafacial bare, at middle 1/2 as wide as the width of postpedicel; antenna black, postpedicel 2.0 times longer than wide, arista black and haired, the longest hair equal to basal aristal width; epistoma not projecting beyond vibrissal angle, vibrissal angle behind frontal angle in profile; subvibrissal setulae in one row, lateral of subvibrissal setulae with one or two rows of fine setae; gena and genal dilation with black setulae, upper margin of gena without upcurved seta; proboscis stout, labella large; prementum with thin gray pollinosity, 1.5 times longer than wide; palpus black, claviform, as long as prementum. Thorax black in ground color, notum slightly shining, with thin brown pollinosity, without distinct stripe; *acr* biserial, slightly stout, prescutellar pairs slightly stout, the distance between *acr* rows 1/2 of the distance between *acr* row and *dc* row, *dc* 2+3, *ia* 0+2, *pra* 1, 4/5 of the length of posterior notopleural seta, notopleuron without setula; proepisternal setae 2, proepimeral seta 1, lower part with one short setula; basisternum, proepisternum, anepimeron, meron and katepimeron bare; katepisternal setae 1+1, katepisternum without ventral spine, with only eleven long setulae on lower margin; anterior spiracle yellowish, posterior one brown; calypters yellowish, the lower one distinctly projecting beyond the upper one. Wing brownish; veins brownish; wing base being same color as rest of the wing; tegula black; basicosta yellowish; costal spine inconspicuous; node of Rs bare on ventral and dorsal surfaces; vein R_4+5_ straight, veins R_4+5_ and M_1+2_ parallel to each other distally; crossveins without obvious cloud; haltere yellow. Legs entirely black, except joint of femur and tibia brown; fore coxa without anterior spine on ventral surface, fore femur with complete *pv* row, fore tibia without *ad* and median *p*, with only a stout seta in preapical part, fore first tarsomere with several longish basal setae on ventral surface; mid coxa without a hook-like spine or spine-like seta, mid femur with long and sparse *av* row in basal half, the longest seta equal to the width of mid femur, becoming gradually denser and shorter towards apex in distal half, comb-like in preapical part, *pv* row complete, slightly biserial in median part, with slender *p* row, mid tibia slightly swollen towards apex, with two *ad* and two *pd*, and with numerous slender setulae on ventral surface, in distal half 3/4 as long as mid tibial width, mid first tarsomere without basal tooth-like spine on ventral surface; hind coxa bare on posterior surface, hind femur with *av* row, only three to five *av* in distal half, stout, *pv* row hair-like in basal half, stout and longer than tibial width in distal half, hind tibia with two *av* (sometimes three), three *ad* (sometimes two) and one median *d*, with several slightly erect median setae on posterior surface, hind tarsi without basal tooth-like spine. Abdomen long and flattened, black in ground color, shining, with thin gray pollinosity; syntergite 1+2 to tergite 4 each with a dark median triangular stripe, tergite 5 with a broad median stripe; sternite one with setulae, sternite 5 broad and rounded, slightly concave on posterior margin, with four strong setulae above (Fig. 4C); cercal plate with a strong curved projection pointed anteriorly in lateral view, bare in median part and at apex (Fig. 4A, B); surstylus broad, separated into two branches at middle, anterior branch broad and with three setulae at apex and posterior branch short and thin (Fig. 4A, B); bacilliform sclerite curved (Fig. 4A, B).

FEMALE. Unknown from China.

##### Remarks.

The species *Fannia norvegica* Ringdahl, 1934 is newly recorded from China. Here, a detailed redescription is provided as it was not been adequately described previously. Illustrations of male terminalia including sternite 5 is also given.

##### Material examined.

1 male, China, Ningxia, Jingyuan, Mt. Heshangpu, 2000 m, 23.VI.2008, Coll. M.F. Wang (IESNU); 1 male, China, Ningxia, Jingyuan, Mt. Heshangpu, 2150 m, 24.VI.2008, Coll. Y.X. Wu (IESNU); 1 male, China, Ningxia, Jingyuan, Mt. Baiyun, 2300 m, 28.VI.2008, Coll. M.F. Wang (IESNU).

##### Distribution.

China: Ningxia, Jingyuan; Czech Republic, Denmark, Great Britain, Greek, Japan, North Africa, Norway, Spain, Switzerland, throughout North America.

## Supplementary Material

XML Treatment for Fannia fani

XML Treatment for Fannia nitidiventris

XML Treatment for Fannia submaculata

XML Treatment for Fannia norvegica
